# Weighted Polynomial Approximation for Automated Detection of Inspiratory Flow Limitation

**DOI:** 10.1155/2017/2750701

**Published:** 2017-05-28

**Authors:** Sheng-Cheng Huang, Hao-Yu Jan, Tieh-Cheng Fu, Wen-Chen Lin, Geng-Hong Lin, Wen-Chi Lin, Cheng-Lun Tsai, Kang-Ping Lin

**Affiliations:** ^1^Department of Electrical Engineering, Chung Yuan Christian University, Taoyuan, Taiwan; ^2^Department of Physical Medicine and Rehabilitation, Chang Gung Memorial Hospital, Keelung, Taiwan; ^3^Graduate Institute of Clinical Medical Sciences, College of Medicine, Chang Gung University, Taoyuan, Taiwan; ^4^Technology Translation Center for Medical Device, Chung Yuan Christian University, Taoyuan, Taiwan; ^5^Department of Biomedical Engineering, Chung Yuan Christian University, Taoyuan, Taiwan

## Abstract

Inspiratory flow limitation (IFL) is a critical symptom of sleep breathing disorders. A characteristic flattened flow-time curve indicates the presence of highest resistance flow limitation. This study involved investigating a real-time algorithm for detecting IFL during sleep. Three categories of inspiratory flow shape were collected from previous studies for use as a development set. Of these, 16 cases were labeled as non-IFL and 78 as IFL which were further categorized into minor level (20 cases) and severe level (58 cases) of obstruction. In this study, algorithms using polynomial functions were proposed for extracting the features of IFL. Methods using first- to third-order polynomial approximations were applied to calculate the fitting curve to obtain the mean absolute error. The proposed algorithm is described by the weighted third-order (w.3rd-order) polynomial function. For validation, a total of 1,093 inspiratory breaths were acquired as a test set. The accuracy levels of the classifications produced by the presented feature detection methods were analyzed, and the performance levels were compared using a misclassification cobweb. According to the results, the algorithm using the w.3rd-order polynomial approximation achieved an accuracy of 94.14% for IFL classification. We concluded that this algorithm achieved effective automatic IFL detection during sleep.

## 1. Introduction

Respiratory event identification is necessary for diagnosis and treatment of sleep-disordered breathing (SDB), including obstructive sleep apnea/hypopnea syndrome (OSAHS), upper airway resistance syndrome (UARS), and snoring. These three conditions comprise the entire SDB spectrum, ranging from the most severe to the mildest [1, 2]. The American Academy of Sleep Medicine states that a subject may snore without apnea or hypopnea occurring. Several studies [3–5] have demonstrated that UARS involves a milder degree of upper airway obstruction than obstructive sleep apnea (OSA). UARS is thought to occupy an intermediate position between the two extremes. Before OSA occurs, most patients experience the UARS stage [6]. Crucially, patients with UARS may eventually progress to OSA if untreated [7].

Patients with UARS are characterized by upper airway collapse (apnea-hypopnea index ≤ 5 events per hour) or more than 30% of total sleep time with inspiratory flow limitation (IFL) during sleep. In recent years, IFL events have been recognized as a vital diagnostic tool for mild SDB [8, 9]. According to the Stanford Sleep Clinic scoring rule [10], for diagnosing mild SDB, at least four successive breaths must be characterized by an abnormal wave contour. Hence, the identification of IFL is essential. Because IFL results from a partial occlusion of the upper airway during sleep, previous studies [3, 11] have reported that IFL events are more difficult to detect than sleep apnea and hypopnea events. Manual scoring of abnormal respiratory events is a time-consuming task. A qualitative grading by visual inspection is more demanding than merely identifying IFL cycles [12]. Therefore, achieving automatic IFL recognition is a crucial task that necessitates resolution in clinical practice.

For automatic noninvasive assessment of IFL and related changes in UARS or OSA, the recognition of both airflow and intrathoracic pressure changes is required; this has been described in previous studies [13, 14]. Various mathematical approaches have been proposed for reliably classifying IFL breaths using the esophageal pressure-flow relationship [2, 14–16]. Recently, several mathematical methods based on the peak, flatness, and roundness of the flow-time curve have been proposed for detecting IFL for autoadjusting positive airway pressure (auto-PAP) devices [17–19]. The theoretical considerations on the basis of which the relationship between flow and time can be characterized using a polynomial equation are presented in [16]. They demonstrate that a 3rd-order polynomial model is appropriate to fit an actual inspiratory flow. On the basis of this study, we hypothesized that a weighted polynomial approximation can effectively achieve classification of IFL.

This study investigated a real-time feature extraction algorithm for detecting IFL during sleep. We propose IFL detection methods involving weighted polynomial functions for IFL feature extraction. For comparative study, both the flatness index (FI) method and polynomial approximation were used for IFL detection. Vital insights into the ability of the proposed methods to automatically detect IFL were thereby obtained.

## 2. Methods

### 2.1. Data Collection and Signal Preprocessing

#### 2.1.1. Development Set

**Figure 1:**
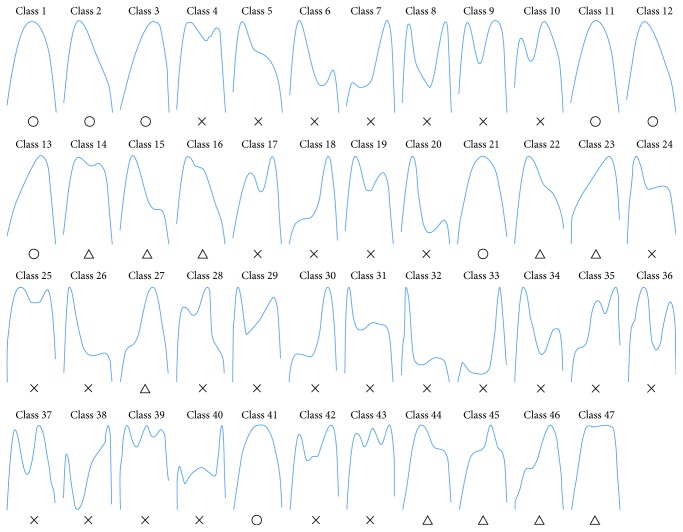
The 47 inspiratory flow data sets used in this study. Non-IFL (normal), IFL Level 1 (mild), and IFL Level 2 (severe) are labeled as “○,” “△,” and “×,” respectively.

**Figure 2:**
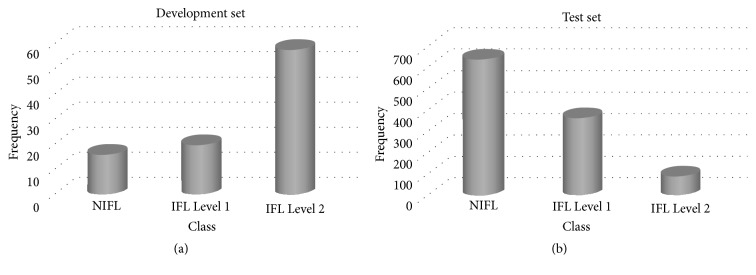
Class distributions determined by the experts. (a) Development set. (b) Test set.

To evaluate the potential feature extraction metrics (or indicators) of IFL and to determine the best cut points for each metric, a development data set was obtained from previous studies [17, 20] Clustering analysis was performed as described in the following section. Aittokallio et al. (2007) classified inspiratory airflow waveforms as non-IFL (normal), IFL Level 1 (mild), and IFL Level 2 (severe), which are labeled as “○,” “△,” and “×,” respectively, in Figure 1 [17]. The data were collected from normal male subjects, normal female subjects, and male patients with OSAHS. The representative patterns of inspiratory flow shape were sorted into Classes 1–10, Classes 11–20, and Classes 21–40 using cluster analysis. Aittokallio et al. (2001) described seven categories, in which Classes 41–43 represented the classes with one, two, or three or more peaks; Classes 41 and 47 differentiate the sinusoidal class and the flat top flow limitation class; and Classes 44–46 provide further differentiation of the single-peak shapes regarding the presence and orientation of the flat part [20]. To quantify the degree of the midinspiratory flattening of the flow-time curve, we collected 47 classes of inspiratory flow shapes to obtain typical and representative single-period waveforms. These served as fundamental evidence of the extent of the three major inspiratory flow waveforms and provided a basis for estimation for characteristic analysis. Data created by time inversion were also collected. Thus, the learning samples consisted of inspiratory flow shapes from 94 breaths and were used to tune the parameters of our proposed algorithm. The distribution is illustrated in Figure 2(a).

#### 2.1.2. Test Set

Thirteen volunteers with OSAHS were recruited from the community. These volunteers underwent full nocturnal polysomnography (Alice® 5 Sleep System Advances Through Simplification, Philips) recordings using EEG, ECG, EOG, EMG, and Spo2 derived at a sample frequency of 256 Hz at the sleep center of Taipei Veterans General Hospital in Taipei, Taiwan. The study was approved by the Institutional Review Board of Taipei Veterans General Hospital, Taiwan (2012-11-001AC). Sleep stages were manually scored by experts using Rechtschaffen and Kales criteria. Apneas and other respiratory events were scored by applying standard criteria [21]. The respiratory signal was obtained using a nasal cannula device at a sampling frequency of 20 Hz and a low-pass filter with a cut-off frequency of 2 Hz was applied. Because the frequency content of a flow signal is located below 1 Hz [22], the applied 2 Hz filter can reduce the signal noise from snoring. In this study, inspiratory flow signals were used to detect flow limitation; the determination and normalization of inspiratory cycles are essential for auto-PAP devices. The procedure was as follows: (1) The actual respiratory flow signal provided by the nasal cannula device was recorded during sleep. Because breaths are connected in inspiratory-expiratory pairs, the expiratory parts were filtered to retain the inspiratory parts for IFL detection. The start of inspiration was marked by a switch from negative to positive flow (relative to the baseline). (2) Each inspiratory flow waveform amplitude was normalized to a range between 0 and 1. (3) Each inspiratory flow waveform duration was normalized to an interval between 0 and 1, with a total of 100 points. All respiratory flow waveforms were preprocessed according to the previously described normalization process.

Respiratory event-related arousal (RERA) is believed to be the characteristic event of OSA. Our test set was obtained from 13 OSA subjects either with or without RERA while sleeping. Their breathing cycles were analyzed as follows. Polysomnography was applied for prescoring to detect each subject's breathing—for 150 seconds prior to RERA or a nonrespiratory-event, and for 150 seconds subsequently, thereby obtaining a section containing non-IFL and IFL breathing cycles. A set of 2565 respiratory data was obtained from the 72 sections collected during sleeping. Subsequently, 1093 respiratory data items were selected from the set through subjective visual detection. Processing of visual detection was conducted as follows. For the separate processing of the non-IFL and IFL data, two physicians with more than 3 years of clinical experience assigned levels of 1–3 in accordance with their respective experience. We used three levels of certainty in our interviews, namely, (1)* strongly negative*, (2)* balanced*, and (3)* strongly positive*. The aim was to not exclude the influence of subjective judgment. Each physician was asked to judge the inspiratory flow following visual assessment and to note the IFL degree as a certainty value. The percentage of agreement was 89.3% between both physicians. The test set created with classical non-IFL criteria consisted of 637 breaths and that with the IFL criteria consisted of 456 breaths (IFL Level 1 (363) and IFL Level 2 (93)), resulting in a total of 1,093 breaths being obtained by strong positive certainty. The distribution is illustrated in Figure 2(b).

### 2.2. Feature Extraction for IFL Detection

#### 2.2.1. Flatness Index

**Figure 3:**
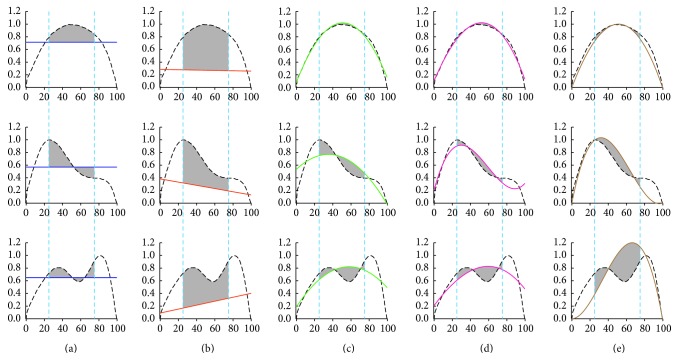
Comparison of the feature extraction methods for non-IFL (top), IFL Level 1 (middle), and IFL Level 2 (bottom). Curve-fitting analysis shows the flow-time curve (dashed line) and the fitting curve (solid line). The residuals between 25% and 75% for the inspiratory flow are indicated by the gray area. (a) FI. (b) First-order polynomial equation. (c) The order of the equation is increased to a second-degree polynomial. (d) The order of the equation is increased to a third-degree polynomial. (e) The w.3rd-order polynomial approximation.

A patented FI process [23] was used to calculate the inspiratory flow of patients during sleep to determine whether their respiration was abnormal. FI is used to treat SDB in various positive airway pressure devices [24]. It is calculated by the root-mean-square deviation from a unit-scaled flow, which is computed over the middle (50%) of a normalized inspiratory breath. A characteristic flattened flow-time curve indicates the highest resistance and the presence of flow limitation, whereas a rounded flow-time curve indicates lower resistance and the absence of flow limitation. A detected indication can acquire a value through the FI formula shown in the following:

(1) $$\begin{array}{c} \text{Flattening Index}=\frac{\sum_{25\%}^{75\%}\left(fs-M\right)}{M*D}, \end{array}$$

where *fs* is the inspiratory flow signal, *M* is the mean of the inspiratory flow, and *D* is the number of breaths. In this study, the value of *D* is 1 because the flow-time curve relates only to a single breath. Flatness is determined by the absolute value of variance between 25% and 75% for the inspiratory flow from the average of all values in the same period. FI detects IFL at 50% of the middle area of an inspiratory signal. A normal inspiratory flow-time curve is rounded or quasi-sinusoidal. For this reason, it is easy to determine whether an inspiratory breath is normal or limited. Examples of FI detection are shown in Figure 3(a).

#### 2.2.2. Polynomial Approximation

To achieve rapid convergence and efficiency, the Levenberg-Marquardt (L-M) method was achieved for obtaining least-squares coefficient solutions. The polynomial models were used to fit the inspiratory flow data and to calculate the residues as feature extraction. Using the residuals, a receiver operator curve was produced to determine the threshold of non-IFL, IFL Level 1, and IFL Level 2 classifications.

*Polynomial Approximation*. The inspiratory flow signal may be in compliance with a *k*-order linear equation [16, 25, 26]:

(2) $$\begin{array}{c} S\left(\;i\;\right)=a_{k}\cdot i^{k}+\cdots +a_{2}\cdot i^{2}+a_{1}\cdot i+a_{0}. \end{array}$$

The polynomial function *S*(*i*) is of exact degree *k* if it is of degree *k* and *a*_*k*_! = 0, where the flow index has the following mathematical representation:

(3) $$\begin{array}{c} R=\overset{y}{\underset{i=x}{\sum }}\left|\;r\left(\;i\;\right)\;\right|=\overset{y}{\underset{i=x}{\sum }}\left|\;S\left(\;i\;\right)-F\left(\;i\;\right)\;\right|. \end{array}$$

Here, *R* is the residual of feature extraction, *r*(*i*) is the residual of the *i*th data point for the total points, *F*(*i*) is the measured inspiratory flow, and *S*(*i*) is the fitted response value. *S*(*i*) is a *k*-order linear equation; *a*_0_, *a*_1_, *a*_2_, *a*_3_, and *a*_*k*_ are constants; *x* is the starting point of the sampling points; *y* is the ending point of the sampling points; *i* is each sampling point between *x* and *y*; and *F*(*i*) is the amplitude of the inspiratory flow at the sampling point *i*.

*Weighted Polynomial Approximation*. For our proposed method, the reference waveform may be in compliance with a *k*-order weighted linear equation *S*(*i*) wherein the feature index has the following mathematical representation:

(4) $$\begin{array}{c} R=\overset{y}{\underset{i=x}{\sum }}W\left(\;i\;\right)\times \left|\;S\left(\;i\;\right)-F\left(\;i\;\right)\;\right|. \end{array}$$

Here, *R* is the residual of feature extraction and *W*(*i*) is defined as

(5) $$\begin{array}{c} W\left(\;i\;\right)=\left\{\begin{array}{cc} A, & i=x,y\\ B, & i=\max\left(F\left(i\right)\right),\\ C, & others, \end{array}\right. \end{array}$$

where *S*(*i*) is the *k*-order linear equation, *x* is the starting point of the sampling points; *y* is the ending point of the sampling points; *i* is each sampling point between *x* and *y*; *W*(*i*) is a weighting function;* A*,* B*, and *C* are weighting factors; and *F*(*i*) is the amplitude *A* of the normalized waveform at the sampling point *i*.

Compared with the fitting effect of the *k*-order linear equation, the *k*-order weighted linear equation can further weight, for example, the starting point *x* or the ending point *y* of the normalized flow curve. This could adjust the end points of the curve from the *k*-order weighted linear equation and reduce the error caused by the end points. In this study, the weighted *k*-order linear equation cites only the starting and ending points. We suggest that the weighting factor “*A*” ranges from 50 to 100, “*B*” ranges from 200 to 400, and “*C*” is 1. The default values of “*A*” and “*B*” are 50 and 200 units, respectively. The value of the weighting factors was determined from the development set and it did not change from patient to patient or breath to breath.

### 2.3. Statistical Analysis

A receiver operator curve (ROC) was constructed for each index of feature extraction for the development set, and cut points were chosen to optimize the separation among the non-IFL, IFL Level 1, and IFL Level 2 groups. Sensitivity, specificity, and accuracy were then calculated. An optimal cut-off for separating subjects with and without IFL was then selected for each index of feature extraction to simultaneously maximize the sum of sensitivity and specificity. The two “best” metrics (maximum area under the ROC) were chosen from among the evaluated indices. The test set was then evaluated prospectively, using only the best indices.

## 3. Results

### 3.1. Results of Accuracy Analysis

**Figure 4:**
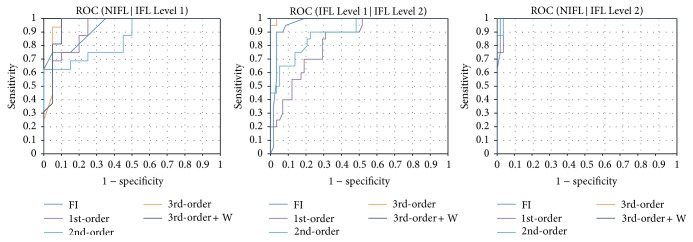
ROC integral curves for the analytical classes. Area Under Curve (AUC) describes the model performance. Predictions of AUC are rated as* acceptable* (>0.7),* excellent* (>0.8), or* outstanding* (>0.9).

According to the curve-fitting results, the flow-time relationships of breaths were classified as non-IFL, IFL Level 1, and IFL Level 2, as shown in Figure 3. The rapid convergence and efficiency were conducted using the L-M method to minimize the sum of the residuals. In this study, IFL detection methods were investigated, as shown in Table 1. The accuracy of the classification of non-IFL, IFL Level 1, and IFL Level 2 in the development set was evaluated through the area under the curve (AUC) of an ROC. Predictions of AUC can be rated as* acceptable* (>0.7),* excellent* (>0.8), or* outstanding* (>0.9).  Figure 2 shows the number of localities in each analytical class. The feature detection methods using the third-order and the weighted third-order (w.3rd-order) polynomial approximations demonstrated outstanding distinction performance for IFL classes. The feature detection methods using FI, the first-order polynomial approximation, and the second-order polynomial approximation provided excellent distinction for the IFL classes. According to the AUC shown in Figure 4, the best cuff-off values were available to classify the three IFL categories, as presented in Table 2. The experimental results of the development set are shown in Table 3. The classification of the non-IFL part—excluding the method of FI detection, which reached only 81.25%—could be trained to 93.75% accuracy. In the classification of the IFL Level 1 part, the detection methods using FI, the first-order polynomial approximation, and the second-order polynomial approximation could be trained up to only 70% accuracy. In the classification of the IFL Level 2, excluding the detection methods using the first-order and 2nd-order polynomial approximations, all feature detection methods could be trained to more than 96% accuracy. Overall, the w.3rd-order polynomial approximation could reach 96.81% accuracy; furthermore, the third-order polynomial approximation reached up to 94.68% accuracy. Thus, we found that the feature detection methods using the 3rd-order and w.3rd-order polynomial approximations achieved much higher accuracy than the other feature detection methods.

The results of test sets for the five types of feature detection methods are shown in Table 4. The overall classification performance indicates that the feature detection method using the w.3rd-order polynomial approximation achieved the best accuracy (94.14%). Subsequently, the feature detection method using the third-order polynomial approximation achieved up to 89.66% accuracy. The FI feature detection method achieved only 78.68% accuracy. The feature detection methods using FI and the first-order polynomial approximation for non-IFL for the analysis of 1,093 breaths exhibited high accuracy (96.70% and 98.90%, resp.), but the accuracy for IFL classification was insufficient. Therefore, this result displays poor recognition for IFL classification. Moreover, IFL was unrecognized in the feature detection methods using the first-order and second-order polynomial approximations, which ultimately displayed accuracy levels of 29.75% and 27.82%, respectively. The sensitivity (true positive rate) and specificity (true negative rate) for each classification are also shown in Tables 3 and 4.

### 3.2. Comparison of Three-Class Classification Performance

**Figure 5:**
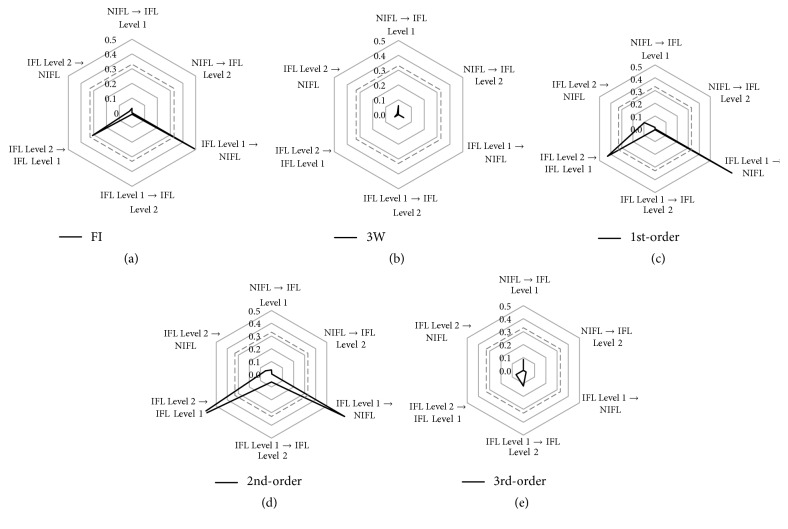
Comparison of three-class misclassification rates for different feature extraction methods. The cobweb plot enables an intuitive comparison of the misclassification rates. Each axis ranges from 0 at the center (no misclassification) and extends outward to 1.0 (100% misclassification) and has been cropped from 0 to 0.5. For example, the axis “IFL Level 2  →  IFL Level 1” refers to the percentage of subjects who were originally IFL Level 2 but misclassified as IFL Level 1 using the feature extraction method. The performances of each feature obtained from different methods are plotted in different colors. (a) FI, (b) w.3rd-order, (c) first-order, (d) second-order, and (e) third-order polynomial approximations.

Cobweb representation is a method of performance assessment for multiclass classification [27]. Using a graphical representation in which each of the separate misclassifications represents one corner on a polygon, a visualization is used to analyze multiclass medical data [28]. In this work, cobweb representation based on the total number of misidentified breaths was used as comparative method of assessing three-class classification performance. The proportion of accurately classified and misclassified samples obtained from the test set is presented in Table 4. The misclassified samples are used in cobweb representation. A chance classification with three classes represented with 1/3 (0.33) likelihood is given in Figure 5. The likelihood is generally used to compare the performance of any classifier with the chance classifier in terms of misclassification rates. In this study, we used cobweb representation to visualize the performance of three-class classifiers on the test set. The performance evaluation shown in Figure 5 is a summary of the comparison of all five feature detection methods. As shown in the figure, the feature detection methods using the third-order and w.3rd-order polynomial approximations exhibited favorable performance. The six-dimensional points of the cobweb are less than 0.1 and less than the chance classifier. However, the feature detection method using the w.3rd-order polynomial approximation is superior to that using the 3rd-order polynomial approximation with respect to (IFL Level 1 → IFL Level 2) misclassification. We also observed that the detection methods using FI, the first-order polynomial approximation, and second-order polynomial approximation showed higher accuracy than did the chance classifier in terms of (IFL Level 1 → NIFL) and (IFL Level 2 → IFL Level 1) misidentification.

## 4. Discussion

The detection of IFL is important in clinical practice involving SDB. Detecting the existence of IFL could not only verify diagnoses of SDB but also enable the supply of urgent airflow support to eliminate flow limitation and treat SDB. This study demonstrated that a real-time feature extraction algorithm based on previous feature classification and using weighted 3rd–order polynomial approximation can effectively identify IFL with a correction rate of up to 96.81%. This is unequivocally helpful in managing SDB. Regarding the automatic detection of the shape of airflow signals, auto-PAP devices have been used for several years to correct the flattened inspiratory flow in order and associate it with subtle upper airway obstruction [29, 30]. Although auto-PAP devices are costlier than standard continuous PAP devices, they are useful for eliminating adverse respiratory events and improving subjective restfulness in specific situations such as home titration and detection of mouth leak. Auto-PAP devices constantly monitor a person's breathing and adapt to the best pressure setting throughout the night. However, auto-PAP technology relies on the determination of obstructive events characterized by IFL, apneas, and snoring to automatically adjust to different pressure settings. The pressure adjustment between low and high ranges is key to the success of auto-PAP devices in improving treatment adherence in patients with SDB.

This study used two indicators—accuracy and misclassification—to assess classification performance. The main finding of this study is that the feature detection methods using FI and the first- and second-order polynomial approximations are weak. In addition, the feature detection methods using the third-order and w.3rd-order polynomial approximations were compared. Although the classification results were highly similar, the feature detection method using the w.3rd-order polynomial approximation achieved superior classification accuracy to that achieved by the method using the third-order polynomial approximation for IFL Level 1. In cobweb representation, the results reveal that the FI method, first-order polynomial approximation, and second-order polynomial approximation yield highly similar results. All exhibit misclassification in the arm of [IFL1 → IFL2] and [IFL1 → NIFL]. The reasons for this may be that (1) the FI method and first-order polynomial approximation applying 50%–60% standard deviation of the IFL middle waveform as a characteristic value and (2) using the second-order polynomial approximation can only indicate a turning point. It may describe a non-IFL waveform but can describe only the trend in the IFL waveform. That is, it cannot act as effective data of flow-limited breath for improving the classification. The second-order polynomial approximation is characterized by only one deflection. In other words, it does not approximate as closely as the first-order approximation (see Figure 3 for details). However, the third-order polynomial approximation is expected to provide a more accurate estimation of the flow-time relationship than the second-order polynomial approximation does for flow-limited breaths because it can be characterized by two deflections. Moreover, a two-deflection relationship can approximate the better measured flow-limited breaths in Levels 1 and 2. Therefore, in this study, a polynomial approximation combined with the weighted principal component is presented as a more effective method for classifying IFL waveforms. The polynomial approximation describes the flow-limited breaths as well as fixing at both ends of the data points and the maximum value with combination of the weight of the principal component. The application of auto-PAP to UARS is a suitable example of the effectiveness of our algorithm in improving auto-PAP. Currently, all types of auto-PAP devices are used in providing optimal ventilation therapy to SDB patients in clinical treatment. The key to effective treatment is firmware programming for assessing the degree of upper airway resistance [31]. UARS is often ignored in classical polysomnographic diagnostic approaches and misdiagnosed as simple snoring or idiopathic hypersomnia. There is still considerable disagreement on the type and level of respiratory abnormality that must be used as a “cut-off” for significant diseases (e.g., OSA syndrome and UARS). In a previous study [32], auto-PAP titration was applied to diagnose and treat patients with UARS to treat daytime sleepiness or somnolence. A useful noninvasive predictor of increased upper airway obstruction has been presented by the evaluation of the inspiratory flow shape. As mentioned, UARS is characterized by repeated arousal at night. However, we know that decreasing upper airway resistance is effective against UARS for patients treated with auto-PAP. The auto-PAP machine could reduce arousal. Hence, it must be assumed that the auto-PAP algorithm accurately detects the presence of flow limitation. Our proposed identification and classification of IFL can be used to facilitate the application of auto-PAP in early treatment. We are confident that it is easy to use and sufficient to detect IFL when using auto-PAP. In summary, our algorithm can assist auto-PAP in providing correct and effective pressure to prevent REMS or severe respiratory obstruction.

Previous studies by Aittokallio et al. [17, 20] have described an automated method using inspiratory flow shape clustering for monitoring the functioning of the upper airway during sleep. In contrast, a reference training set for IFL data using this summarization approach was successfully applied in our work. This appears to be the first time that a reference set has been used to develop a model for estimating IFL. Furthermore, IFL shapes were classified into different levels (non-IFL Level 1, IFL Level 2 (mild), and IFL Levels 3 and 4 (severe)) [18, 19]. In our study, the shapes of IFL were classified into just two levels. Mansour et al. [25, 26] demonstrated a polynomial equation for determining flow limitation based on the pressure-flow relationship. In the present study we applied the w.3rd-order polynomial function using the flow-time relationship. Several limitations should be considered in interpreting our findings. First, the inspiratory flow shapes were visually classified breath-by-breath on one occasion. The test set involved a low number of actual respiratory flow shapes. The results of the sensitivity and specificity analysis were not compared with other functions, such as the quadratic function [25]. Finally, IFL with inspiratory snoring was not included in the analysis data.

## 5. Conclusion

In conclusion, the third-order algorithm demonstrated high performance for IFL detection. The w.3rd-order polynomial function significantly improved the population and was successfully applied for classification of severe IFL. This comparative study provides vital insights into the efficacy of automatic detection of IFL for reducing mean airway pressure, improving subjective sleepiness, and ensuring adherence to treatment in patients with mild SDB.

## Figures and Tables

**Table 1:** Comparison of the area under the ROC curve (AUC). Predictions of AUC are considered acceptable (>0.7), excellent (>0.8), or outstanding (>0.9).

Two-class	FI	1st-order	2nd-order	3rd-order	w.3rd-order
ROC (NIFL ∣ IFL level 1)	0.838	0.934	0.856	0.953	0.948
ROC (IFL level 1 ∣ IFL level 2)	0.906	0.822	0.884	0.998	1.000
ROC (NIFL ∣ IFL level 2)	0.989	0.991	0.996	1.000	1.000

**Table 2:** Cut-off points of classification criteria.

Class∖method	FI	1st-order	2nd-order	3rd-order	w.3rd-order
NIFL	>0.175	>6.19	≤1.17	≤0.34	≤0.87
IFL Level 1	>0.095 & ≤0.175	>4.54 & ≤6.19	>1.17 & ≤1.96	>0.34 & ≤1.02	>0.87 & ≤2.7
IFL Level 2	≤0.095	≥4.54	>1.96	>1.02	>2.7

**Table 3:** Overall classification results of the development set (*n* = 94). Accuracy, sensitivity = true positive rate (TP), and specificity = true negative rate (TN) are considered.

Methods	Measure	NIFL	IFL Level 1	IFL Level 2	Total
FI	% correct	81.25%	70.00%	96.55%	88.30%
	Correct #	13	14	56	83
	TP/TN	0.72/0.96	0.77/0.92	0.97/0.94	—

1st-order	% correct	93.75%	65.00%	68.97%	72.34%
	Correct #	15	13	40	68
	TP/TN	0.68/0.98	0.43/0.89	0.95/0.65	—

2nd-order	% correct	93.75%	40.00%	77.59%	72.34%
	Correct #	15	8	45	68
	TP/TN	0.56/0.98	0.40/0.84	0.96/0.72	—

3rd-order	% correct	93.75%	90.00%	96.55%	94.68%
	Correct #	15	18	56	89
	TP/TN	0.94/0.99	0.86/0.97	0.98/95	—

w.3rd-order	% correct	93.75%	90.00%	100.00%	96.81%
	Correct #	15	18	58	91
	TP/TN	0.88/0.98	0.95/0.97	1.0/1.0	—

**Table 4:** Performance results of the test set (*n* = 1,093). Accuracy, sensitivity = true positive rate (TP), and specificity = true negative rate (TN) are considered.

Methods	Measure	NIFL	IFL Level 1	IFL Level 2	Total
FI	% correct	96.70%	50.41%	65.59%	78.68%
	Correct #	616	183	61	860
	TP/TN	0.77/0.93	0.75/0.79	0.97/0.97	—

1st-order	% correct	98.90%	29.75%	43.01%	71.18%
	Correct #	630	108	40	778
	TP/TN	0.70/0.97	0.68/0.73	0.93/0.95	—

2nd-order	% correct	88.85%	27.82%	69.89%	66.97%
	Correct #	566	101	65	732
	TP/TN	0.70/0.75	0.69/0.72	0.73/0.97	—

3rd-order	% correct	91.52%	85.40%	93.55%	89.66%
	Correct #	583	310	87	980
	TP/TN	0.98/0.89	0.84/0.93	0.66/0.99	—

w.3rd-order	% correct	93.56%	93.56%	96.77%	94.14%
	Correct #	596	343	90	1029
	TP/TN	0.98/0.91	0.89/0.97	0.94/0.99	—
